# An audit of endodontic diagnostic documentation compliance among undergraduate dental students in a UK primary care–based dental school

**DOI:** 10.1186/s12909-026-08676-z

**Published:** 2026-02-03

**Authors:** Araz Ahmed, Casper Jonker, Marina Bozic, Ewen McColl

**Affiliations:** Peninsula Dental School, University of Plymouth, Plymouth, UK

**Keywords:** Audit, Endodontics, Undergraduate dental education, Diagnostic documentation, Diagnostic reasoning

## Abstract

**Background:**

Erroneous pulpal and periapical diagnoses can lead to inappropriate treatment planning, unnecessary procedures, and compromised patient outcomes. While undergraduate endodontic education emphasises history taking, clinical testing and radiographic assessment, less is known about how consistently students document diagnostic conclusions in clinical records.

**Aim:**

To evaluate the completeness and compliance of endodontic diagnostic documentation among undergraduate dental students, benchmarked against British Endodontic Society (BES) and European Society of Endodontology (ESE) standards, and to explore trends across different stages of training.

**Methods:**

Records of 196 root canal treatments completed by Year 3–5 Bachelor of Dental Surgery students (2022–2024) were reviewed. Audit standards were based on the British Endodontic Society Guide to Good Endodontic Practice (2022) and United Kingdom record-keeping guidance. Parameters included history-taking, sensibility testing, radiographic justification and reporting, and documentation of provisional and definitive diagnoses. Compliance was analysed descriptively, with limited inferential comparison where appropriate.

**Results:**

Compliance with core record keeping elements was high across all cohorts. Documentation of diagnostic conclusions and signs and symptoms varied across student groups and academic years. Provisional diagnoses were recorded in 12.37% of cases overall, whereas definitive diagnoses were documented in 66.31%, with the highest compliance observed in Year 5. Documentation of signs and symptoms was 65.83% in 2022–2023, increasing to 67.76% in 2023–2024.

**Conclusions:**

Undergraduate dental students consistently document clinical findings relevant to endodontic diagnosis; however, explicit recording of diagnostic conclusions, particularly provisional diagnoses, and documentation of signs and symptoms remain inconsistent. Strengthening educational emphasis on documenting diagnostic reasoning may support preparedness for independent practice.

## Introduction

Dental students must develop both clinical and non-clinical competencies during their undergraduate education to make informed decisions on patient care [1]. Diagnosing pulpal and periapical diseases is challenging, and errors at this stage can have undesirable consequences. An erroneous diagnosis may lead to inadequate treatment planning, superfluous procedures, or unsatisfactory patient outcomes [2].

Initial exposure to endodontics typically occurring later in the second year of the Bachelor of Dental Surgery (BDS) course at Peninsula Dental School (PDS). Undergraduates create endodontic access and perform cleaning, shaping and obturation on single and multi-rooted artificial teeth (incisors and premolars) in the Simulated Dental Learning Environment (SDLE). To align the practical skills and science behind endodontic disease, endodontics is taught from the second year onwards through a series of lectures, workshops and chairside teaching on clinics. To progress to the third year, students are required to successfully pass an endodontic capability assessment. Once students’ progress to their third year, they repeat the process but this time focusing on artificial molars. Students also typically start treating patients endodontically in the third year. Importantly, and in common with other dental schools, PDS undergraduates are taught the principles of endodontic diagnosis, including identification of signs and symptoms and the use of sensibility testing to support clinical decision-makings [1, 2].

A precise diagnosis in endodontics requires a systematic approach, beginning with a comprehensive history, followed by pertinent diagnostic evaluations, and culminating in a treatment plan that considers the long-term prognosis of the tooth [1, 3–5]. Challenges increase when sensibility test results sometimes conflict with the patient’s presenting symptoms, underscoring the necessity of robust preclinical and clinical abilities to facilitate decision-making during training and subsequent independent practice [3, 6–8].

Current research demonstrates insufficient evidence concerning endodontic diagnostic processes and documentation in undergraduate education, despite a limited number of studies assessing diagnostic competence among dental undergraduates. One questionnaire-based study reported that advanced training enhances diagnostic accuracy, underscoring the importance of clinical experience and diagnostic confidence [2]. Nevertheless, this study was limited to simulated environments and did not evaluate student performance with real patients.

The predominant focus of reported United Kingdom audits in endodontics has been on evaluating the technical quality of student root canal procedures rather than the documentation of diagnostic processes [9–12]. This focus has facilitated enhancements in education across both primary care based and secondary care based dental schools. Research undertaken in Dublin, Belfast, and Plymouth has analysed radiographic and technical outcomes among undergraduate students [9–12]. However, these studies have not explicitly evaluated undergraduate compliance with standards for documenting pulpal and periapical diagnoses that inform treatment decisions, indicating a need for further investigation in this area.

The General Dental Council (GDC) requires undergraduate students to demonstrate diagnostic competence in endodontics, as specified in *‘The Safe Practitioner’*, ensuring that graduates can assess, diagnose, and develop treatment strategies for pulpal and periapical diseases [6]. Likewise, The European Society of Endodontology (ESE) and the British Endodontic Society (BES) undergraduate curriculum guidelines stipulate that dental graduates must demonstrate competence in acquiring dental histories, performing pulp sensibility assessments, analysing radiographs, and developing diagnoses to inform appropriate treatment planning [4, 13]. A previous PDS audit evaluating the technical quality of undergraduate root canal treatments reported outcomes comparable to other UK and Irish secondary care based dental schools, suggesting that favourable results can be achieved in a primary care-based educational setting [9]. Building on this foundation, the present audit evaluates undergraduate compliance with endodontic diagnostic documentation standards across different stages of training, using BES and ESE guidance as benchmarks.

## Materials and methods

A retrospective audit was conducted on 196 root canal treatments performed by Year 3, 4, and 5 undergraduate students at PDS during 2022–2024. Cases were stratified by tooth type: 60 anterior and 136 posterior teeth. Treatments were performed under routine clinical supervision. Patient records were selected randomly by a trained dental nurse, independent of the treating students. Data extraction and analysis were undertaken by a consultant in restorative dentistry, a clinician with special interest in endodontics, and a senior oral surgery speciality trainee, all blinded to student identity. Ethical approval was obtained from the University of Plymouth, and data collection followed the General Data Protection Regulation (GDPR) guidelines.

Audit standards were derived from the BES *Guide to Good Endodontic Practice* (2022) and UK record-keeping guidance (Faculty of General Dental Practice (FGDP), College of General Dentistry (CGDent) and NHS England), covering history-taking (including the SOCRATES framework), sensibility testing, radiographic justification and reporting, and the documentation of provisional and definitive diagnoses [4, 14–19]. Because no gold-standard diagnosis or independent validation was applied, the outcomes of this audit reflect compliance with diagnostic documentation standards rather than diagnostic accuracy. The parameters assessed included:

- Student year and tooth type.
- History of trauma.
- Clinical tests performed (percussion, sensibility, mobility, radiographs).
- Signs and symptoms documented (pain, sensitivity, sinus tract, discolouration, swelling, fractures).
- Radiographic/periapical findings.
- Provisional and definitive diagnosis documentation (BES categories: RP, IP, PN, TAP, SAP, AAP, AAA, CAA, CO).

Compliance with standards was calculated as the proportion of cases meeting benchmarks, assessed by three independent, blinded reviewers (Table 1).

**Table 1:** Audit standards and benchmarks

Parameter	Standard / Benchmark	Target	Notes
Student year (BDS level)	Accurate recording of the year of training	100%	Ensures correct stratification for audit
Tooth type treated	Accurate documentation (anterior/posterior)	100%	Based on clinical notes
History of trauma	Recorded (present/absent; details if present)	100%	Per BES guidelines
Clinical tests undertaken	Must include appropriate tests to support diagnosis	100%	E.g., sensibility testing, percussion, palpation, mobility, periodontal assessment, radiographs
Signs and symptoms	Must be documented fully	100%	Use SOCRATES for pain history; record associated features (e.g., pain on biting, swelling, sinus tract, discolouration, fracture/restoration issues)
Radiographic/periapical findings	Must record presence/absence of pathology	100%	E.g., PDL widening, lamina dura changes, radiolucency/radiopacity
Provisional diagnosis	Must be recorded at initial endodontic assessment	100%	
Definitive diagnosis	Must be recorded before or at initiation of endodontic treatment	100%	BES diagnostic categories: RP, IP, PN, TAP, SAP, AAP, AAA, CAA, CO

### Statistical analysis

Statistical analysis was primarily descriptive. Where appropriate, a two-sided unpaired Student’s *t*-test was used to compare overall compliance rates between provisional and definitive diagnosis documentation, with significance set at *p* < 0.05. Overall compliance for provisional and definitive diagnosis documentation was calculated using a weighted mean to account for differences in cohort size (Year 3 *n* = 25, Year 4 *n* = 71, Year 5 *n* = 100; total *N* = 196). Weighted compliance was calculated by multiplying each cohort’s compliance percentage by the number of students in that cohort, summing these values, and dividing by the total number of students.

## Results

**Fig. 1:**
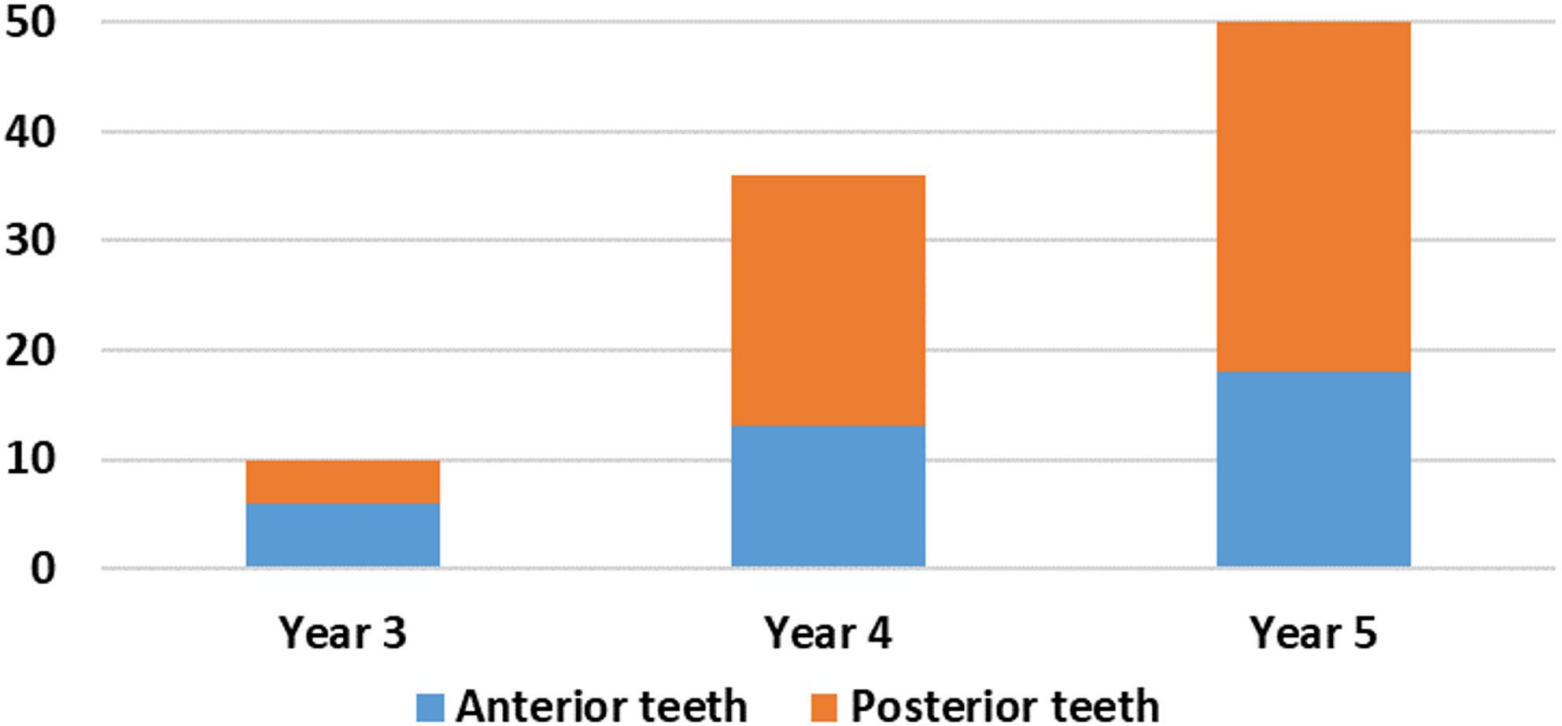
Distribution of root canal treatments by year group (2022-2023)

**Fig. 2:**
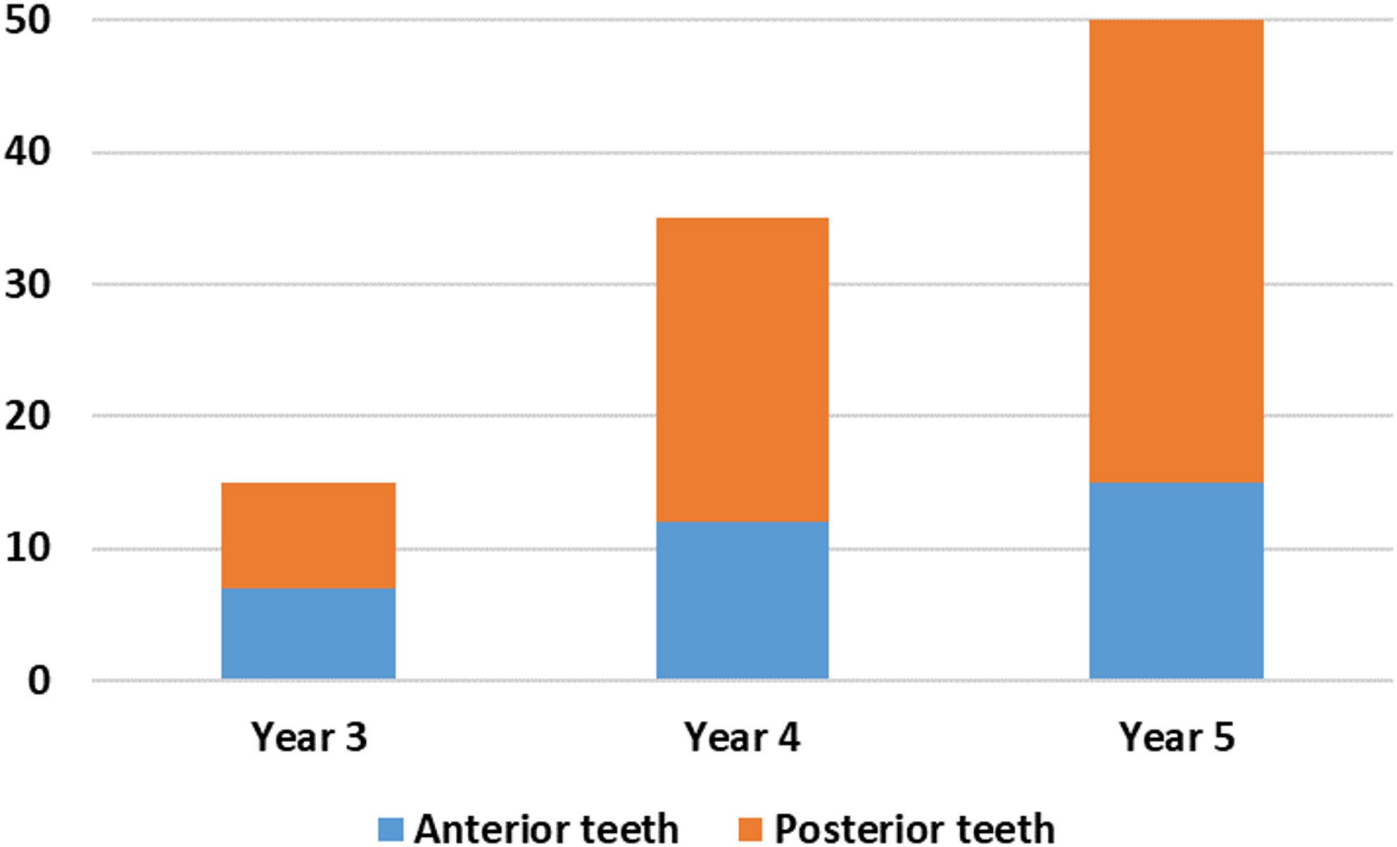
Distribution of root canal treatments by year group (2023-2024)

A total of 196 root canal treatments were evaluated, comprising 96 cases from the 2022–2023 academic year and 100 cases from 2023 to 2024. Of these, 71 treatments involved anterior teeth (36.22%), while 125 involved posterior teeth (63.78%). Treatments were undertaken by Year 3 (*n* = 25), Year 4 (*n* = 71), and Year 5 (*n* = 100) undergraduate students. In both academic years, the majority of cases involved posterior teeth, accounting for 63% of cases in 2022–2023 (Fig. 1) and 66% in 2023–2024 **(**Fig. 2**)**.

**Fig. 3:**
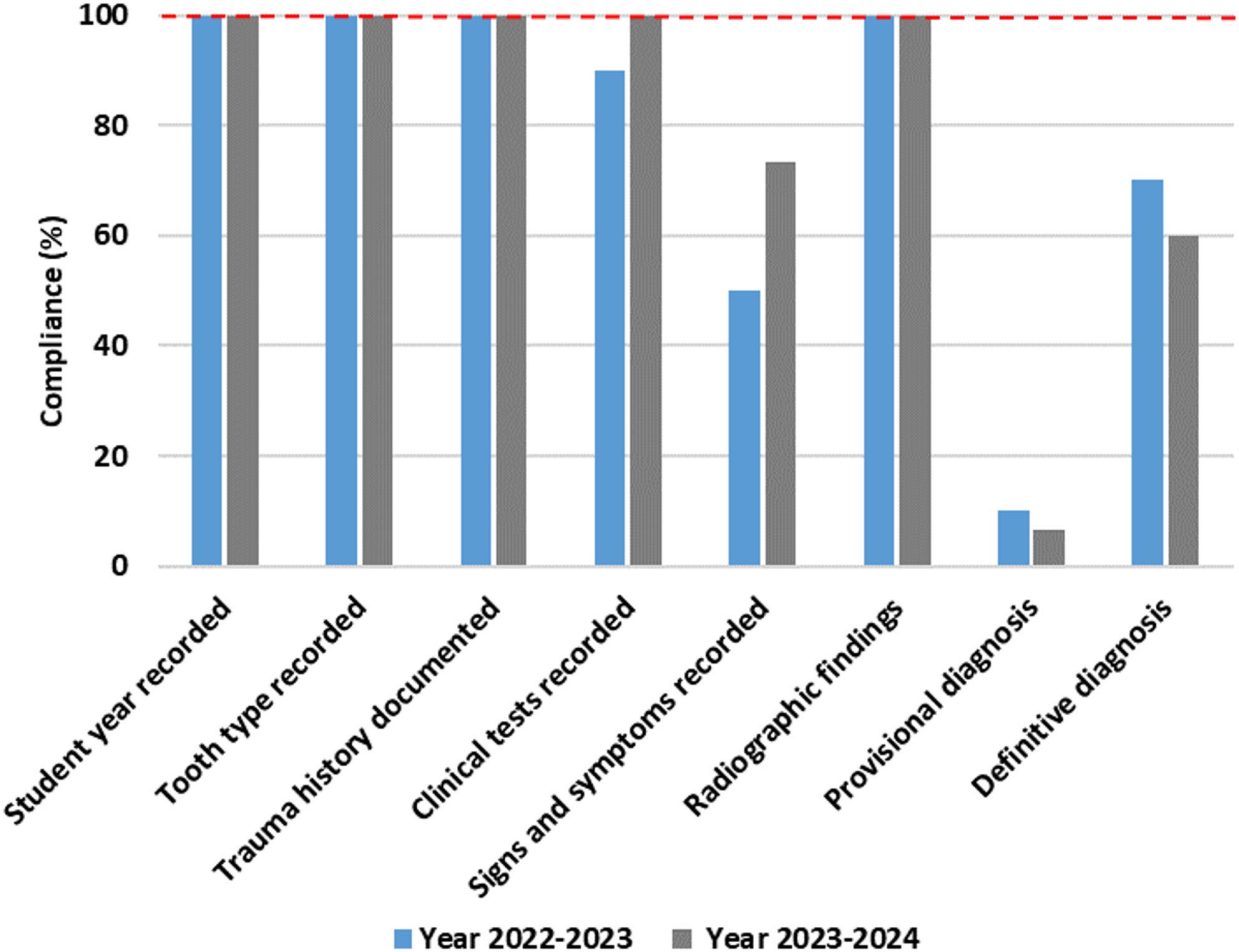
Year 3 compliance (2022-2024)

**Fig. 4:**
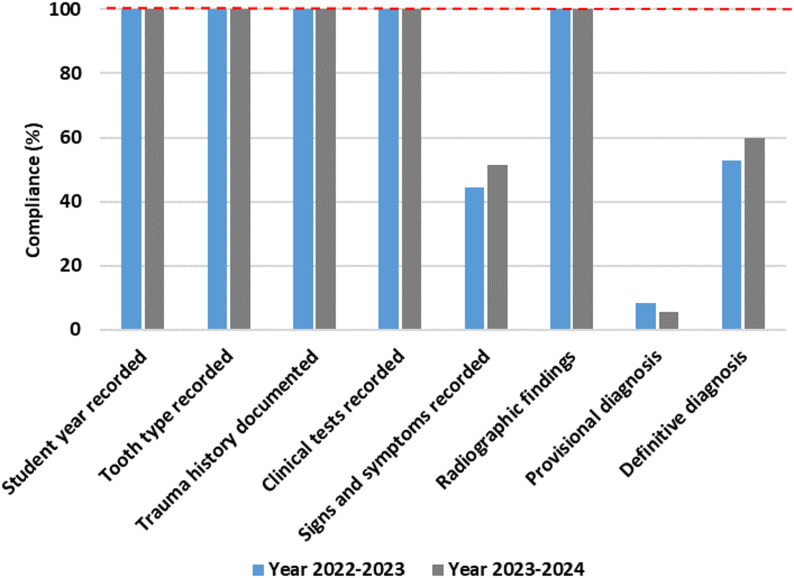
Year 4 compliance (2022-2024)

**Fig. 5:**
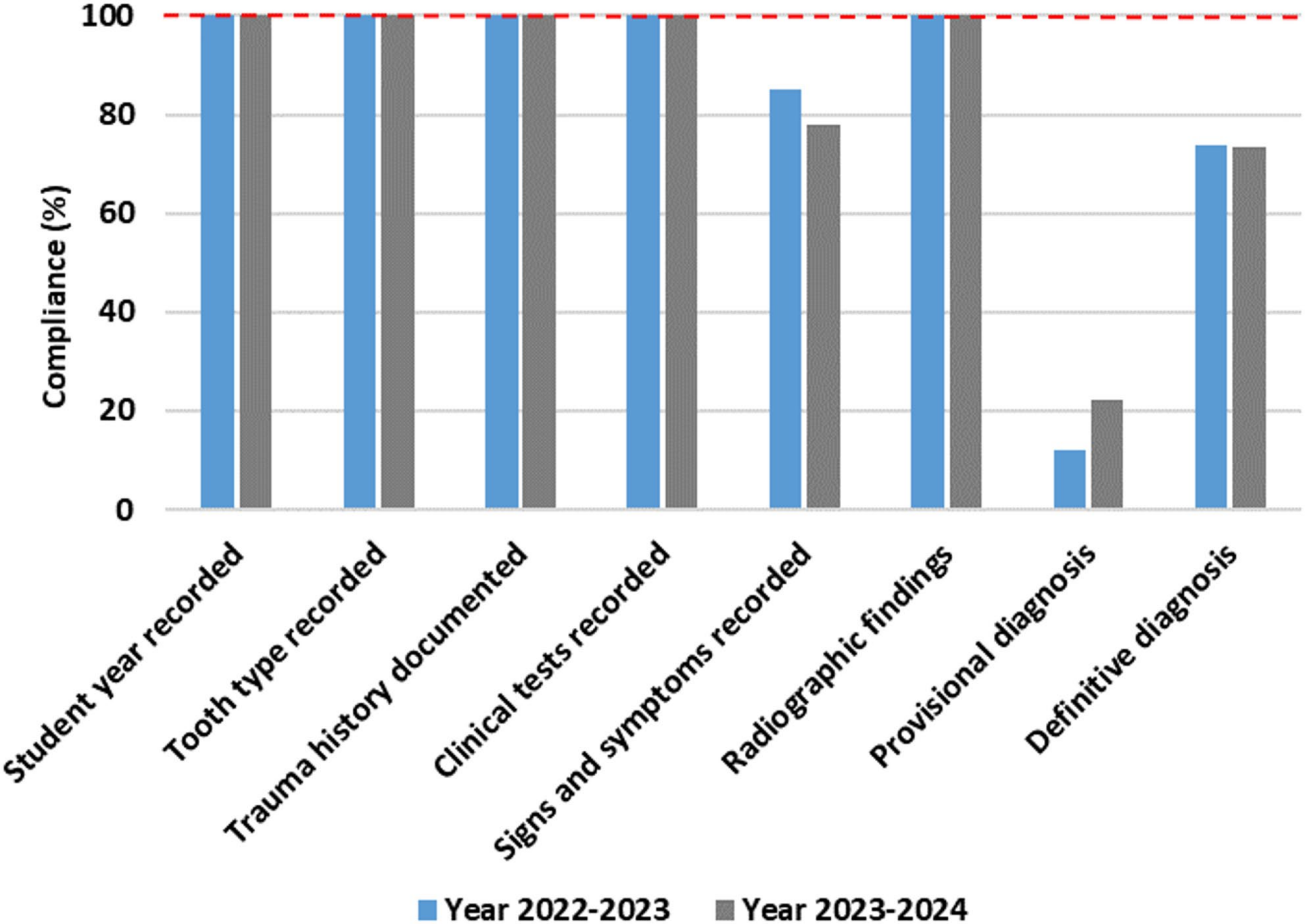
Year 5 compliance (2022-2024)

The cases treated by Year 5 students reached the highest levels in both years, with 52% in 2022–2023 and 50% in 2023–2024, indicating a notable advancement in clinical exposure and skill proficiency. Across both academic years, adherence to fundamental record-keeping standards was high. All cases included complete documentation of student year, tooth type, trauma history, clinical tests, and radiographic findings for Year 3 (Fig. 3), Year 4 (Fig. 4), and Year 5 students (Fig. 5), demonstrating consistent compliance with procedural documentation requirements.

In contrast, documentation related to diagnostic conclusions and signs and symptoms varied across cohorts and academic years. Among Year 3 students, provisional diagnoses were documented in 10% of cases in 2022–2023 and 6.6% in 2023–2024, while definitive diagnoses were recorded in 70% and 60% of cases, respectively. Documentation of signs and symptoms was observed in 50% of cases in 2022–2023, increasing to 73.3% in 2023–2024.

Year 4 students recorded signs and symptoms in 44.4% of cases in 2022–2023 and 51.4% in 2023–2024, remaining below Year 3 in both academic years. Documentation of provisional diagnoses remained low, recorded in only 8.3% and 5.7% of cases across the two academic years respectively. Documentation of definitive diagnoses was recorded in 52.7% of cases in 2022–2023 and 60% in 2023–2024.

Year 5 students demonstrated the highest compliance with definitive diagnosis documentation, recorded in 74% of cases in 2022–2023 and 73.4% in 2023–2024. Despite this, documentation of provisional diagnoses remained limited, recorded in 12% of cases in 2022–2023 and 22.4% in 2023–2024. Documentation of signs and symptoms was recorded in 85% of cases in 2022–2023 and 78% in 2023–2024.

**Fig. 6:**
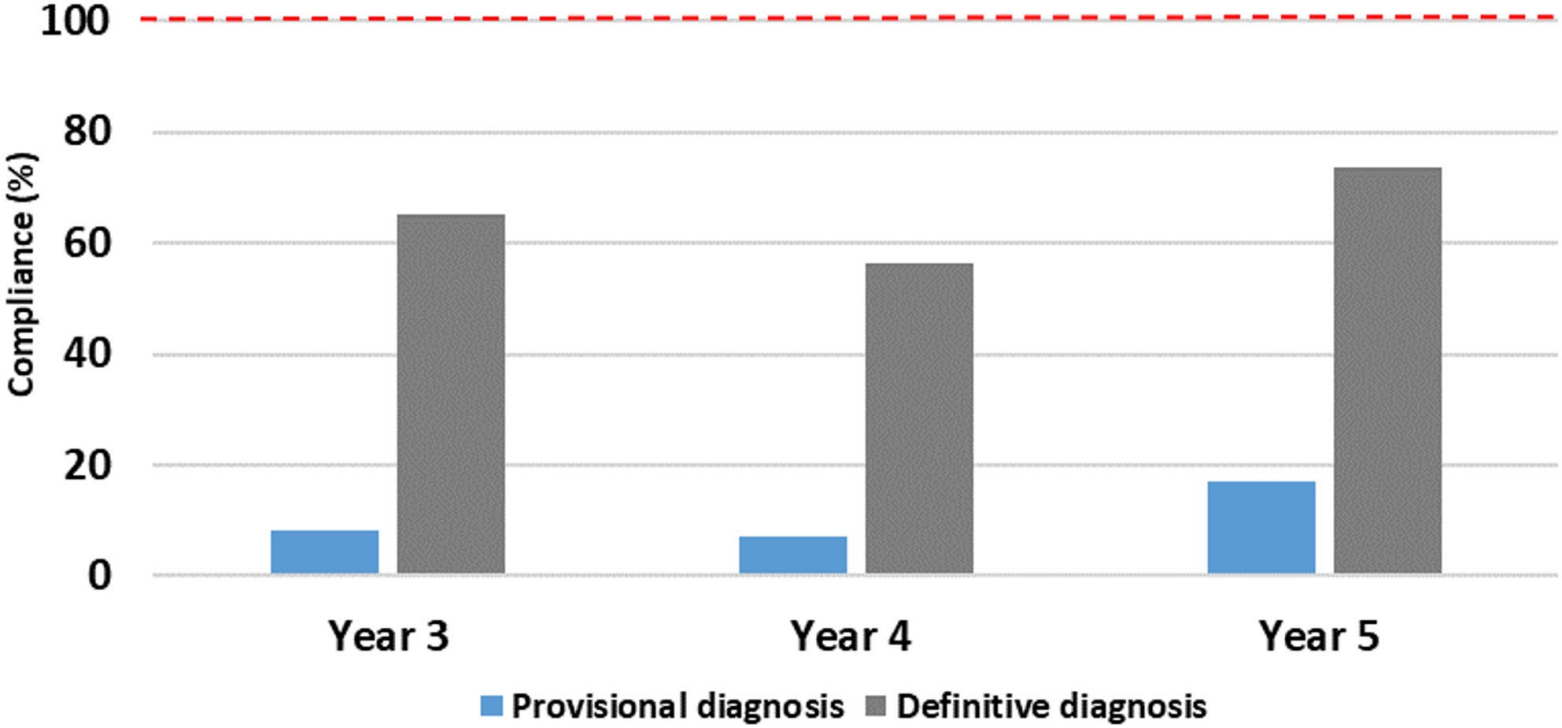
Mean provisional and definitive diagnosis compliance by year group (2022–2024)

When data from both academic years were combined, mean compliance for documentation of provisional diagnoses remained consistently low across all student groups. In contrast, mean compliance for definitive diagnosis documentation was higher overall but varied across student cohorts, with the highest compliance observed in Year 5; however, differences between groups were not consistent across academic years **(**Fig. 6**)**.

**Fig. 7:**
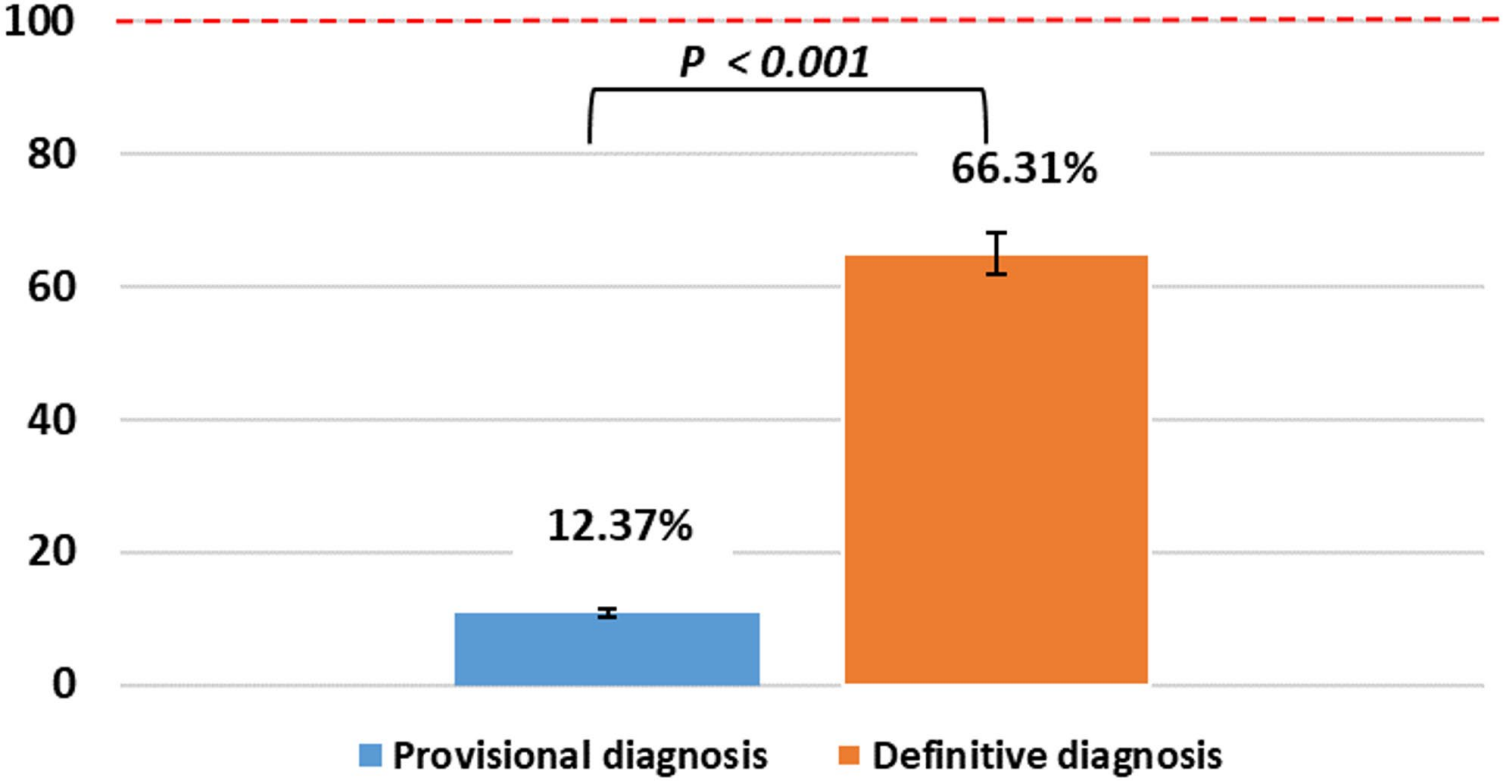
Mean compliance: Provisional vs Definitive diagnoses (2022-2024)

A comparison of weighted mean compliance rates across all student years (2022–2024) demonstrated a statistically significant difference between provisional and definitive diagnosis documentation (Fig. 7). When weighted by cohort size (Year 3 *n* = 25, Year 4 *n* = 71, Year 5 *n* = 100), overall compliance for provisional diagnosis documentation was 12.37% ± 7.02, compared with 66.31% ± 9.52 for definitive diagnosis documentation (t = 10.10, *p* < 0.001). Similarly, the weighted mean compliance rate for signs and symptoms documentation across cohorts increased from 65.83% in 2022–2023 to 67.76% in 2023–2024; however, compliance remained below the audit benchmark.

## Discussion

This audit evaluated compliance with endodontic diagnostic documentation standards among undergraduate students at Peninsula Dental School (PDS) across two academic years (2022–2024). Core record-keeping was exemplary, with all cohorts consistently documenting student year, tooth type, history of trauma, and radiographic investigations. These findings indicate strong adherence to procedural and record-keeping requirements and alignment with General Dental Council (GDC), European Society of Endodontology (ESE), and British Endodontic Society (BES) expectations [4, 6, 13, 20]. However, documentation of signs and symptoms did not meet the audit benchmark of 100% compliance and demonstrated variable performance across cohorts and academic years.

To interpret these findings, it is important to outline the educational and clinical context in which undergraduate endodontics is delivered at PDS. Peninsula Dental School is located in the Southwest of England and is considered one of the “Dental Deserts” of the UK [21]. While patients in these regions face challenges accessing dental treatment under the National Health Service (NHS), this setting provides valuable educational opportunities. Patients can access dental care delivered by undergraduates at four Dental Educational Facilities (DEFs) located in Derriford (Plymouth), Exeter, Devonport and Truro [21]. Undergraduate students are supervised by highly skilled and experienced clinical supervisors during clinical sessions. At PDS, students receive early patient exposure from their first year of dental studies through a primary care-based model [9], undertaking a range of treatments from an early stage, including patient examinations and placement of simple restorations under strict rubber dam isolation.

As part of this model, all dental students at PDS must demonstrate competence in a series of endodontic skills within the Simulated Dental Learning Environment (SDLE) before progressing to patient care. The practical component is carefully interwoven with academic teaching (lectures and workshops) and is delivered through a spiral curriculum from Year 2 to Year 5, designed to promote increasing understanding and critical thinking [22]. The curriculum progresses from the correct application of rotary instrumentation in Year 3, to non-complicated endodontic retreatment in Year 4, and more specialised techniques such as heated obturation and reciprocation in Year 5. Across successive years, students are also exposed to increasingly complex diagnostic scenarios, including teeth restored with full coverage restorations, sclerotic pulpal networks and traumatic dental injuries. The benefits of this spiral approach to dental education at PDS have been well documented [23].

Within this educational context, the present audit provides insight into the consistency with which undergraduate students document endodontic diagnostic conclusions across different stages of training. The principal deficit identified was the explicit recording of diagnostic conclusions. Although appropriate clinical tests were performed and documented, both provisional and definitive diagnoses were recorded inconsistently across all year groups, with provisional diagnoses rarely documented. The relatively small Year 3 sample introduces imprecision, and these findings should therefore be interpreted with caution. Documentation of definitive diagnoses was higher in Year 5 compared with Years 3 and 4; however, compliance remained below the audit benchmark and should not be interpreted as indicating a linear improvement across cohorts [24, 25].

In addition, documentation of signs and symptoms was variable and remained below the expected standard across all years. Although documentation increased in the later academic year, it did not reach the benchmark of full compliance, indicating an area for improvement in the recording of patient-reported findings and clinical presentation.

The observed variation between Year 3 and Year 4 cohorts may reflect transitional factors, including increased clinical workload, greater case complexity and a shift from more scaffolded learning to more independent clinical practice, rather than a regression in diagnostic capability. Although this audit included both anterior and posterior teeth, it was not designed to evaluate the effect of tooth type on diagnostic documentation compliance. Differences in case complexity between anterior and posterior teeth may plausibly influence documentation practices; however, further evaluation would be required to explore this relationship.

These findings are consistent with the literature describing the complexity of endodontic diagnosis, which requires integration of subjective patient-reported symptoms with objective clinical and radiographic findings that may be discordant [1, 5, 25]. While the present audit did not examine concordance between symptoms and diagnostic tests, existing evidence provides important context for understanding the challenges faced by students during diagnostic decision-making. Although higher documentation rates were observed in more senior cohorts, particularly Year 5, these patterns should be interpreted cautiously due to the imbalance in sample sizes across cohorts and the absence of a consistent stepwise progression between years.

From an educational perspective, the findings suggest that undergraduate students are consistently collecting appropriate diagnostic information, particularly clinical test results and radiographic findings, but may benefit from greater emphasis on translating this information into clearly documented diagnostic conclusions. Similarly, incomplete documentation of signs and symptoms suggests that students may require additional support in systematically recording the clinical presentation and symptom history as part of the diagnostic process. Importantly, the absence of documented provisional diagnoses does not necessarily indicate an absence of diagnostic reasoning, as diagnostic discussions may occur verbally during clinical supervision or be influenced by local documentation practices and record templates, and because documentation may preferentially capture final (definitive) diagnostic conclusions rather than interim (provisional) formulations.

To address these gaps, targeted educational measures are planned to strengthen diagnostic documentation practices. These include structured teaching on the interpretation of sensibility tests and radiographs, supervisor-facilitated reflective discussions during clinics that require students to articulate and record provisional and definitive diagnoses, and incorporation of diagnostic documentation outcomes within formative and summative assessments. Further emphasis will also be placed on structured recording of signs and symptoms, to reinforce the link between the clinical presentation and diagnostic decision-making. Such measures align with ESE undergraduate curriculum guidance, BES standards, and preparation for independent practice [1, 4, 5, 7, 13, 24, 25].

In summary, undergraduate students at PDS demonstrate strong compliance with documenting clinical and investigative components of endodontic assessment; however, consistent documentation of diagnostic conclusions requires further emphasis. Documentation of signs and symptoms also remained below the audit benchmark, indicating scope for improvement in recording patient-reported and clinical presentation data. To our knowledge, this is the first audit conducted within a primary care-based dental school to evaluate undergraduate compliance with endodontic diagnostic documentation standards. The findings support continued evaluation in comparable educational settings, and similar audits across UK dental schools may help benchmark documentation practices and inform curriculum development. A re-audit is planned following implementation of the educational measures to evaluate their impact on diagnostic documentation compliance.

## Data Availability

The datasets used and/or analysed during the current study are available from the corresponding author on reasonable request. The dataset is derived from retrospective clinical educational records and is not publicly available due to confidentiality and data protection requirements.

## Abbreviations

AAA: Acute Apical Abscess
AAP: Asymptomatic Apical Periodontitis
BDS: Bachelor of Dental Surgery
BES: British Endodontic Society
CAA: Chronic Apical Abscess
CGDent: College of General Dentistry
CO: Condensing Osteitis
DEF: Dental Educational Facility
ESE: European Society of Endodontology
FGDP: Faculty of General Dental Practice
GDPR: General Data Protection Regulation
GDC: General Dental Council
IP: Irreversible Pulpitis
PDS: Peninsula Dental School
NHS: National Health Service
PN: Pulpal Necrosis
RP: Reversible Pulpititis
SAP: Symptomatic Apical Periodontitis
SDLE: Simulated Dental Learning Environment
SOCRATES: Site, Onset, Character, Radiation, Associations, Time course, Exacerbating/relieving factors, Severity
TAP: Transient Apical Periodontitis
UK: United Kingdom
